# Understanding the predictive value of continuous markers for censored survival data using a likelihood ratio approach

**DOI:** 10.1186/s12874-019-0721-0

**Published:** 2019-05-22

**Authors:** Andrew M. Smith, John P. Christodouleas, Wei-Ting Hwang

**Affiliations:** 1 Department of Biostatistics, Epidemiology, and Informatics, 610 Blockley Hall 423 Guardian Drive, Philadelphia, 19104 USA; 2 Department of Radiation Oncology, 3400 Civic Center Boulevard, Philadelphia, 19104 USA; 3 3250 Whitfield Ave Apt 211, Cincinnati, OH, 45220 USA

**Keywords:** Survival data, Biomarker, Likelihood ratio, Predictive value, Landmark analysis, ROC

## Abstract

**Background:**

The likelihood ratio function (LR), the ratio of conditional probabilities of obtaining a specific marker value among those with the event of interest over those without, provides an easily interpretable way to quantify the update of the risk prediction due to the knowledge of the marker value. The LR has been explored for both binary and continuous markers for binary events (e.g., diseased or not), however the use of the LR in censored data has not been fully explored.

**Methods:**

We extend the concept of LR to a time-dependent LR (TD-LR) for survival outcomes that are subject to censoring. Estimation for the TD-LR is done using Kaplan-Meier estimation and a univariate Cox proportional hazards (PH) model. A “scale invariant” approach based on marker quantiles is provided to allow comparison of predictive values between markers with different scales. Relationships to time-dependent receiver-operator characteristic (ROC) curves, area under the curve (AUC), and optimal cut-off values are considered.

**Results:**

The proposed methods were applied to data from a bladder cancer clinical trial to determine whether the neutrophil-to-lymphocyte ratio (NLR) is a valuable biomarker for predicting overall survival following surgery or combined chemotherapy and surgery. The TD-LR method yielded results consistent with the original findings while providing an easily interpretable three-dimensional surface display of how NLR related to the likelihood of event in the trial data.

**Conclusions:**

The TD-LR provides a more nuanced understanding of the relationship between continuous markers and the likelihood of events in censored survival data. This method also allows more straightforward communication with a clinical audience through graphical presentation.

## Background

Biomarkers are measurable characteristics that are used to identify the likelihood of a future event. A common goal of biomarker research is quantifying the ability of proposed markers to predict the event of interest. Primary interest often lies in determining whether, relative to existing knowledge of event likelihood, the marker improves event predictions. Additionally, comparison of markers with respect to predictive value is also of interest.

One method of summarizing predictive value is the likelihood ratio (LR), the ratio of conditional probabilities of obtaining a specific marker value given event status (i.e., with and without event). The LR is a well-accepted and valuable method of evaluating potential markers because it can be shown that the value of the LR summarizes the predictive value of a marker by quantifying the update to the odds of event obtained by incorporating knowledge of the new marker in question. An easily interpretable and intuitive update to the “pre-test” probability of a diagnosis or clinical event obtained from examination findings allows for swift and concise judgment of the value of a specific test. For example, the *Journal of the American Medical Association* includes a long-running series of articles entitled “The Rational Clinical Exam” that focuses on the LR as a measure of predictive value– and, subsequently, a clinical decision making tool– in assorted disease scenarios [1].

Methods of estimating and comparing LR for binary events (e.g., diseased or not) for both binary and continuous markers have been explored, but LR in the case of survival data, where event status may change over time and individuals may be censored, has not been established. Thus we propose the time-dependent likelihood ratio (TD-LR) as a measure for the predictive value of continuous markers under a survival analysis framework. We believe the TD-LR can provide a fuller understanding of the relationship between a marker and the likelihood of an event over time than is given by more common measures like the hazard ratio (HR) from a Cox proportional hazards (PH) model. For example, in survival data analysis, characterizing the probability of an event during short, intermediate, or long timeframes based on present knowledge of a specific biomarker could more intuitively be done with separate values of the TD-LR than with a single hazard ratio value.

Graphical presentations of the TD-LR prove useful in communicating predictive value with clinical investigators and non-statisticians. The TD-LR can also be estimated using scale-invariant techniques, which can satisfy the need to compare predictive value across markers. These methods are illustrated in an application to evaluate the predictive value of the neutrophil-lymphocyte ratio (NLR) as a prognostic marker for overall survival (OS) using data from the Southwest Oncology Group (SWOG) 8710 clinical trial, a randomized phase III trial assessing radical cystectomy (RC) with or without neoadjuvant chemotherapy (NAC) for muscle-invasive bladder cancer [2, 3].

### Likelihood ratio (LR) for binary event

#### Definition of LR

We first review the concept of the likelihood ratio (LR) for a binary event *D* and a marker *X*. Let *D*=1 if the event occurs and *D*=0 otherwise, and let *X* be a marker (either binary or continuous). The LR for a given value of *X* is defined as

1 $$\begin{array}{@{}rcl@{}} \frac{P(X=x | D=1)}{P(X=x | D=0)}. \end{array} $$

To understand the intuition behind using the LR as a measure of predictive value, consider the conditional odds of the event *D* given a marker value *X*=*x*:

2 $$\begin{array}{@{}rcl@{}} \frac{P(D = 1 | X =x)}{P(D=0 | X= x)}. \end{array} $$

Using Bayes’ Theorem, these odds can be reexpressed as a product of the LR as defind above and the prevalence-based or marginal odds of *D*, $\frac {P(D=1)}{P(D=0)}$:

3 $$\begin{array}{@{}rcl@{}}  \frac{P(D = 1 | X =x)}{P(D=0 | X= x)} =\frac{P(X = x | D = 1)}{P(X=x | D = 0)}\times \frac{P(D=1)}{P(D=0)}. \end{array} $$

Thus, the LR can be interpreted as the “update" to the odds of event *D* obtained by incorporating knowledge of the marker value. In other words, the initial likelihood of an event is captured by the odds of the event based only on the prevalence of the event in the population, $\frac {P(D=1)}{P(D=0)}$, before incorporating the marker values and $\frac {P(X = x | D = 1)}{P(X=x | D = 0)}$ afterward. We expect useful or informative markers to be those Xs that dramatically change these prevalence-based odds by incorporating knowledge of the marker value *x*.

Therefore, the LR quantifies this update. It represents the degree to which the prevalence-based odds are adjusted by a given marker value. When the LR is >1, the given marker value is more common in the population experiencing the event (i.e., *D*=1), so the odds based on prevalence are adjusted upward to yield the conditional odds of event given *x*. Similarly, if the LR is <1, the given marker value *x* is observed more frequently amongst the population not experiencing the event (i.e., *D*=0), so the odds based on prevalence are adjusted downward. An LR of 1 indicates that incorporating knowledge of the given marker value provides no update to the prevalence-based odds. Therefore, the marker would not be informative in predicting the event. In this manner, the LR is similar in interpretability to the Bayes factor, where the prevalence-based odds of event are considered the “prior”, and the adjusted odds given knowledge of the marker value are the “posterior”. Kass et al. [4] note that interpretation of the LR is the same for binary or continuous markers *X*.

#### Estimation of LR for binary events

For binary events, methods of estimating the LR have been explored for several marker types [5, 6]. For binary markers (positive or negative) and binary events, marker-positive LR and marker-negative LR can be expressed respectively in terms of true positive rate (TPR) and false positive rate (FPR):

$$\begin{array}{@{}rcl@{}} LR(X=+) &=& \frac{P(X = +| D = 1)}{P(X= + | D = 0)}= \frac{TPR}{FPR}, \\ LR(X=-) &=& \frac{P(X = -| D = 1)}{P(X= - | D = 0)}= \frac{1-TPR}{1-FPR}. \end{array} $$

These LRs are estimated with empirical estimators of the TPR and FPR. Comparison of LRs between two different binary markers is straightforward because markers can only be positive or negative, thus there are no concerns of different marker scales.

Estimation of the LR for biomarkers measures on a continuous scale requires other approaches. Gu and Pepe [7] propose a fleet of methods for estimating continuous marker LR values: one that uses the ratio of nonparametric Gaussian kernel estimators for the density of the marker in event and non-event populations, and one that models log*LR*(*x*) as the difference of logits. In case-control data, logit*P*(*D*=1) is fixed, so modeling logit*P*(*D*=1|*X*=*x*) with conventional logistic regression yields an easily obtainable estimator for log*LR*(*x*) with desirable properties such as consistency and asymptotic normality. Comparison of LR estimates across markers with different scales is also explored through making transformation of markers to a standardized scale.

## Methods

The cases of binary or continuous markers for a binary event encompass many applications, but fail to address a common clinical setting. In survival analysis context, interest lies not only in whether or not an event occurs, but in how long it takes to occur. Individuals may also be censored, such that they do not experience the event of interest during the observation period. Existing estimation methods for LR do not directly account for changing event status over time or censoring, and thus require new approaches.

### Notation

We use the following notation throughout this section. Let *T*_*i*_ and *C*_*i*_ denote failure time and censoring time for *i*th individual. Let *δ*_*i*_ be an event indicator equal to 1 if *T*_*i*_≤*C*_*i*_ and 0 otherwise. Denote the observed survival time as *Z*_*i*_=*min*(*T*_*i*_,*C*_*i*_). Let the counting process *D*_*i*_(*t*)=1 if *T*_*i*_≤*t* and *D*_*i*_(*t*)=0 if *T*_*i*_>*t*; that is, given time *t*, *D*_*i*_(*t*)=1 if individual *i* has an event at or prior to *t*. Finally, let the marker value for the *i*th individual be *X*_*i*_.

### Definition of TD-LR

Recall from (3) that the expression for the odds of a general event *D* conditional on marker value *x* can be expressed as a product of LR and the prevalence-based odds. We can rearrange (3) to obtain an expression of the LR function as a product of conditional event probabilities and the prevalence-based odds:

4 $$\begin{array}{@{}rcl@{}}  LR(x) = \frac{P(X = x | D = 1)}{P(X=x | D = 0)} = \frac{P(D = 1 | X =x)}{P(D=0 | X= x)} \times \frac{P(D=0)}{P(D=1)}. \end{array} $$

We then define the time-dependent likelihood ratio (TD-LR) function at time *t* and marker value *x*, *TD*−*LR*(*x,t*) by replacing *D* with *D*(*t*) in the above expression for *LR*(*x*) as follows:

5 $$\begin{array}{@{}rcl@{}} TD - LR(x, t) = \frac{P(D(t) = 1 | X =x)}{P(D(t)=0 | X= x)} \times \frac{P(D(t)=0)}{P(D(t)=1)}. \end{array} $$

The notation *D*(*t*)=1 denotes the event or the condition of (*T*≤*t*) that is time-dependent. Similarly, the notation of *D*(*t*)=0 denotes event-free, (*T*>*t*). The TD-LR function retains much of the same interpretation as the general LR described in previous sections. It represents the update to the prevalence-based odds of event at or before time *t* obtained through measurement of the marker *X*. The above expression provides appealing flexibility with respect to allowing event status to change over time through *D*(*t*). The TD-LR function allows a marker’s predictive value to change over time; for example, some marker values may be more predictive of events at or before later time points *t* than they are of events at earlier *t*. Most importantly, the TD-LR function can accommodate censoring through proper estimation of *D*(*t*), allowing us to make use of information from individuals censored before the time point of interest. Estimation methods are described next.

### Estimation Methods

To estimate the TD-LR function, we propose to use the Kaplan-Meier nonparametric survival estimator [8] and survival estimates dervied from Cox PH models [9]. For a given *t* and marker value *x*, *TD*−*LR*(*x,t*) can be expressed using survival probabilities because the event of *D*(*t*)=0 or *D*(*t*)=1 represents whether (*T*>*t*) or (*T*≤*t*):

6 $$\begin{array}{@{}rcl@{}} TD - LR(x, t) = \frac{1-S(t |X=x)}{S(t | X= x)} \times \frac{S(t)}{1-S(t)} \end{array} $$

where *S*(*t*)=*P*(*T*>*t*) represents the survival function and *S*(*t*|*X*=*x*)=*P*(*T*>*t*|*X*=*x*) the survival function conditional on marker value *x*.

An estimator for the TD-LR function for a marker value *x* at a given time *t* can be obtained by combining the survival probability estimates from KM, $\hat {S}_{{KM}}(t)$, and Cox PH models, $\hat {S}_{{Cox}}(t | X=x)$:

7 $$\begin{array}{@{}rcl@{}}\widehat{TD - LR(x, t)} = \frac{1-\hat{S}_{{Cox}}(t | X=x)}{\hat{S}_{{Cox}}(t |X=x)} \times \frac{\hat{S}_{{KM}}(t)}{1-\hat{S}_{{KM}}(t)}. \end{array} $$

Let $\mathcal {T}$ be the set of observed failure times in a sample. The Kaplan-Meier (KM) estimator of *S*(*t*) can be expressed as

$$\begin{array}{@{}rcl@{}} \hat{S}_{{KM}}(t) = \prod_{s \in \mathcal{T}, s \leq t} \left(1 - \frac{\sum\nolimits_{i} I(Z_{i} = s)\delta_{i}}{\sum\nolimits_{i} I(Z_{i} \geq s)}\right). \end{array} $$

The estimated survival function conditional on marker value from the Cox PH model can be obtained [10] as:

$$\begin{array}{@{}rcl@{}} \hat{S}_{{Cox}}(t |X=x) = \widehat{S_{0}(t)}^{\exp(\hat{\beta}x)}, \end{array} $$

where $\widehat {S_{0}(t)}=\exp (-\widehat {\Lambda _{0}(t)})$ with $\widehat {\Lambda _{0}(t)}$ is the estimated baseline cumulative hazard function. The estimate $\hat {\beta }$, a regression parameter relating the covariate *x* to the hazard, is obtained through partial likelihood maximization and is in turn used to estimate $\widehat {S_{0}(t)}$ by the method outlined in Kalbfleisch and Prentice [10].

This estimator of TD-LR demonstrates some desirable properties. As a function of two arguments, it can readily be visualized as a three-dimensional surface, providing an intuitive display of how different marker values are associated with updates to event risk over different time points. The ability to construct these surfaces can provide clinical investigators with a better understanding of a marker’s relationship to an event than summary statistics such as HRs.

Additionally, estimates of survival probabilities based on Kaplan-Meier method and Cox PH model are appropriate to use even if there is censoring. Moreover, Kalbfleisch and Prentice Chapter 5.6 outline a proof that under certain regularity conditions (most important that the number of individuals at risk for any *t* becomes large as *n*→*∞*), $\hat {S}_{{KM}}(t)$ is consistent for *S*(*t*) and asymptotically normally distributed for any given *t*. Kalbfleisch and Prentice [10] similarly, Tsiatis provides a proof that $\hat {S}_{{Cox}}(t |X=x)$ is consistent for *S*(*t*|*X*=*x*) and asymptotically normally distributed [11]. Therefore, the proposed estimator $\widehat {TD - LR(x, t)}$ at a given *t* and marker value *x* is thus consistent for *TD*−*LR*(*x,t*) by the continuous mapping theorem [12].

The asymptotic distribution of the proposed estimator or its log-transformed version can also be established by the fact that $\hat {S}_{{KM}}(t)$ and $\hat {S}_{{Cox}}(t | X=x)$ can be rewritten as sum of *n* independent empirical functions [11, 13]. Then by applying the (multivariate) delta method [14], it can be shown that, for example, $\sqrt {n} (\hat {S}_{{KM}}(t)/ \hat {S}_{{Cox}}(t | X=x) - S_{{KM}}(t)/ S_{{Cox}}(t | X=x))$ yields two terms plus a remainder term that goes to 0 in probability; the terms either involve $(\hat {S}_{{KM}}(t)-S_{{KM}}(t))$ or $(\hat {S}_{{Cox}}(t)-S_{{Cox}}(t))$ and therefore are asymptotically normal. Asymptotic normality of $\sqrt {n} \log (\widehat {TD - LR(x, t)})$ is also demonstrated by simulation studies (data not shown) with time points and/or marker values associated with fewer number of events (e.g. early or late *t*) requires larger sample size to achieve normality. Asymptotic variance or standard errors are intractable to derive analytically but can be obtained by using bootstrap. In the “Results” section, we present pointwise bootstrap percentile confidence intervals for the TD-LR surface.

Note that our estimate of the TD-LR function is valid only if the assumptions underpinning the validity of the Cox PH model are met. Namely, the validity of $\widehat {TD - LR(x, t)}$ depends on noninformative censoring, proportional hazards, and linearity of the marker effect on the log hazard. If this assumption does not hold, alternative estimates of $\widehat {TD - LR(x, t)}$ will be needed. For example, one can consider parametric regression models for survival time or the use of accelerated failure time models.

### Scale-invariant estimation methods

#### Placement value

Comparing the TD-LR values from two different markers is not intuitive, primarily because there are rarely natural mappings from the scale of one marker to another that would invite comparison at specific points on those scales. To solve the challenges of differing marker scales, we extend the concept of the placement value from Gu and Pepe [7] to standardize different markers to a single scale. For a marker *X* and binary event *D*, the placement value U(X) is calculated as

8 $$\begin{array}{@{}rcl@{}} U(X) = 1 - F_{D=0}(X), \end{array} $$

where *F*_*D*=0_ is the cumulative distribution function (CDF) for the marker in the non-event population. That is, the placement value represents the proportion of individuals not experiencing the event that have higher marker values than *X*. Under the assumption that higher values of a marker indicate higher risk of event, large marker values correspond to small placement values, and small marker values correspond to large placement values. Note that this assumption that can always be met by transforming marker values (e.g., by negating values).

The placement value is a common standardization method. Its use is motivated by the concept of comparing individuals with an event or condition to a “healthy” reference population, similar in principle to using percentiles to describe certain measurements like height and weight amongst infants [7, 15, 16].

In the binary event case, the reference group used to standardize marker values is comprised of controls (i.e. individuals who do not experience the event of interest). In the survival analysis setting, the reference population for placement value *U*(*x,t*) for marker value *x* at time *t* in the context of the TD-LR is thus the set of individuals surviving beyond *t*:

9 $$\begin{array}{@{}rcl@{}} U(x, t) = 1 - F_{D(t)=0}(x). \end{array} $$

Note, the distribution function *F*_*D*(*t*)=0_(*x*) for the marker *x* is constructed based on the *x* values only for those *D*(*t*)=0, that is, those are still risk set at time *t*. For those had the event before time *t* (i.e., *D*(*t*^′^)=1 for *t*^′^<*t*) or those censored before time t are no longer in this risk set thus not used for estimating *F*_*D*(*t*)=0_(*x*). Thusly defined, placement value in the survival context is a time-dependent covariate. As a result, estimating TD-LR for a given time and marker value now requires estimating survival probabilities from a Cox PH model that includes placement value as a time-dependent covariate.

### Time-dependent covariates

The concepts surrounding time-dependent covariates are reviewed insightfully by many including Cortese and Andersen [17]. There are external and internal time-dependent covariates. The manner in which external covariates *X*(*t*) depend on time is not affected by failures at time *u* for *u*≤*t*. External covariates may be defined, in that their dependence on time can be fully specified in advance for all individuals under study (e.g. age), or ancillary, in that their time dependence relies on a process external to the individuals under study and is unrelated to the parameters in the model under study.

$\hat {S}_{{Cox}}(t | X=x)$ when *X* is an external time-dependent covariate can be obtained in the same fashion as before. Time-dependent covariates that are not external are internal. Internal covariates can typically be characterized as measurements that depend on the individual under study surviving and remaining uncensored, such as blood pressure readings taken at certain intervals over the course of follow-up. More generally, internal covariates are generated by the behavior of the individuals in the study over time, thus the covariate “path” at *t*>*u* is influenced by failures at *u*. As a consequence, the typical relationship between the hazard and survival functions no longer exists [10]. Indeed, it is frequently the case that the survival function for internal time dependent covariates is trivially 1, since valid measurements of the covariate at a given time point require the individual to be alive and uncensored at that time.

### Landmark analysis for scale-invariant TD-LR

The placement value *U*(*x,t*) as we have defined it is considered an internal time-dependent covariate which depends directly on the survival behavior of the individuals under study, and, assuming that higher marker values are associated with higher likelihoods of event, conveys some information about the failure time of the individual. As such, we cannot employ the straightforward method of estimating survival functions from the case of markers on their original scales. Instead, we adopt landmark analysis proposed by van Houewelingen [18] and demonstrated by Cortese and Andersen [17] to address internal time-dependent covariates when survival probabilities (or cumulative incidences, as is the case in Cortese) are of interest.

In landmark analysis, a set of “landmark” times *s* of interest are chosen, and simple Cox PH models are fit for each using only the subset of individuals alive and uncensored at *s*. Time-dependent covariates are fixed at the “new baseline” *s* in each model. Survival probabilities for different values of the now-fixed internal covariate can be compared within a landmark subset, and trends across landmark times are examined to provide insight into how the internal covariate affects the risk of event.

To compute a scale-invariant TD-LR at a given *t* through a landmark approach. Let *u* be the placement value of a given marker value *x* at the given landmark time *s*. The reference population for this placement value is all individuals alive and uncensored (i.e. at risk) at *s*. Within the given landmark subset analysis, *u* remains fixed. The scale invariant TD-LR estimate $\widehat {TD- LR_{{SI}}(u,t|s)}$ can thus be expressed as

10 $$\begin{array}{@{}rcl@{}} \widehat{TD - LR_{{SI}}(u, t | s)} = \frac{1-\hat{S}_{{Cox}}(t | T \geq s, u)}{\hat{S}_{{Cox}}(t | T \geq s, u)} \times \frac{\hat{S}_{{KM}}(t | T \geq s)}{1-\hat{S}_{{KM}}(t | T \geq s)}. \end{array} $$

Note that *u* is time independent. Often, *u* can be further transformed as a standardization technique. The transformation is frequently *Φ*^−1^(1−*u*) [7].

To examine the trend across landmark times, we can calculate and compare values of this estimate at a fixed time forward (e.g., two or three years) from each landmark *s*. An illustration of this technique is provided in the next section

### Relationship to TD-ROC

ROC curves are commonly used to compare the predictive ability of continuous markers for a binary event [19, 20]. In the binary event case, the use of the scale-invariant LR and placement value for standardizing marker values yields a mathematical relationship between ROC curves and the LR [7, 15, 16]. We can show the same relationship holds for the TD-LR and the time-dependent ROC (TD-ROC) introduced by Heagerty et al. [21, 22].

Let *F*_*D*(*t*)=1_(*x*) and *F*_*D*(*t*)=0_(*x*) be the cumulative distribution functions of the marker *X* in the subsets of individuals experiencing the event *D* at or before *t* and those not experiencing the event at or before *t*, respectively, and *f*_*D*(*t*)=1_(*x*) and *f*_*D*(*t*)=0_(*x*) be the corresponding probability density functions for the marker values. Then, by definition,

11 $$\begin{array}{@{}rcl@{}} TD- ROC(r, t) = 1- F_{D(t)=1} \left((1-F_{Dt(\cdot)=0}(r))^{-1}\right), \end{array} $$

where *r* is a given false positive rate. Note that the distribution function *F*(·), density function *f*(·) and the inverse function of 1−*F*(·) are for the marker *X* within the subset of *D*(*t*)=1 or *D*(*t*)=1, not as functions associated with the survival time *T*. Differentiating this expression with respect to *t* using chain rule yields

12 $$\begin{array}{@{}rcl@{}} TD- ROC^{\prime}(r, t) = \frac{f_{D(t)=1}\left(F^{-1}_{D(t)=0}(1-r)\right)}{f_{D(t)=0}\left(F^{-1}_{D(t)=0}(1-r)\right)}. \end{array} $$

Thus, for a marker value *x*, time point *t*, take the false positive rate *r*=*U*(*x,t*)=1−*F*_*D*(*t*)=0_(*x*):

13 $$\begin{array}{@{}rcl@{}} TD- ROC^{\prime}(U(x,t), t) &=& \frac{f_{D(t)=1}\left(F^{-1}_{D(t)=0}\left(1-\left(1- F_{D(t)=0}(x)\right)\right)\right)}{f_{D(t)=0}\left(F^{-1}_{D(t)=0}\left(1-\left(1- F_{D(t)=0}(x)\right)\right)\right)}  \\&=& \frac{f_{D(t)=1}(x)}{f_{D(t)=0}(x)}. \end{array} $$

Rewrite *f*_*D*(*t*)=1_(*x*) and *f*_*D*(*t*)=0_(*x*) as *P*(*X*=*x*|*D*(*t*)=1) and *P*(*X*=*x*|*D*(*t*)=0) then the above expression becomes $\frac {P(X = x | D(t)=1)}{P(X=x | D(t)=0)}$ which can be expressed as *TD*−*LR*(*x,t*) as defined in (5) after applying Bayes’ rule.

That is, at a given time *t* and marker value *x*, *TD*−*LR*(*x,t*) represents the derivative of the corresponding TD-ROC curve when the false positive rate takes the value at placement value *U*(*x,t*). Suppose there are two markers *X*_1_ and *X*_2_, and that *X*_1_ is more informative than *X*_2_. Because a more informative marker will have higher TD-LR for small placement values (large marker values) and lower TD-LR for large placement values (small marker values), the above derived relationships imply that for the TD-ROC derivatives for the markers (indexed as $TD- ROC^{\prime }_{1}$ and $TD- ROC^{\prime }_{2}$) are related as follows:

$$\begin{array}{@{}rcl@{}} TD - ROC^{\prime}_{1}(u, t) &> TD - ROC^{\prime}_{2}(u, t) \quad {u} \text{ small} \\ TD - ROC^{\prime}_{1}(u, t) &< TD - ROC^{\prime}_{2}(u, t) \quad {u} \text{ large}  \end{array} $$

Because TD-ROC curves are always concave and “tied down” at the corners (0,0) and (1,1), these conditions imply *AUC*(*t*) for marker *X*_1_ is greater than *AUC*(*t*) for marker *X*_2_, where *AUC*(*t*) represents area under the TD-ROC curve for the given time *t*. Therefore, we can use scale invariant TD-LR analysis together with the relationship between the TD-LR and TD-ROC derivative to make comparisons of the AUC for two different markers across different time points.

## Results

### Simulation

As previously noted, TD-LR is a function of time *t* and marker value *x*, thus the utility of TD-LR can be understood by visualizing it as a three dimensional surface. To illustrate this, we simulated n=100, 300, 500 exponentially-distributed survival times that are associated with a standard normally distributed marker *X* with hazard ratios (HR) of 1.5 and 2. Censoring times are also assumed to follow exponential distribution with a maximum follow-up of five years. Baseline hazard parameters for the event and censoring hazard functions were set equal to 7 and 10 respectively, typically yielding censoring proportions of between 20 and 30%. The log-transformed TD-LR is then calculated over a grid from 1 to 36 months in increments of 3 months and marker values from -2 to 2 in increments of 0.1. For each HR specification, 500 simulations are conducted.

**Fig. 1:**
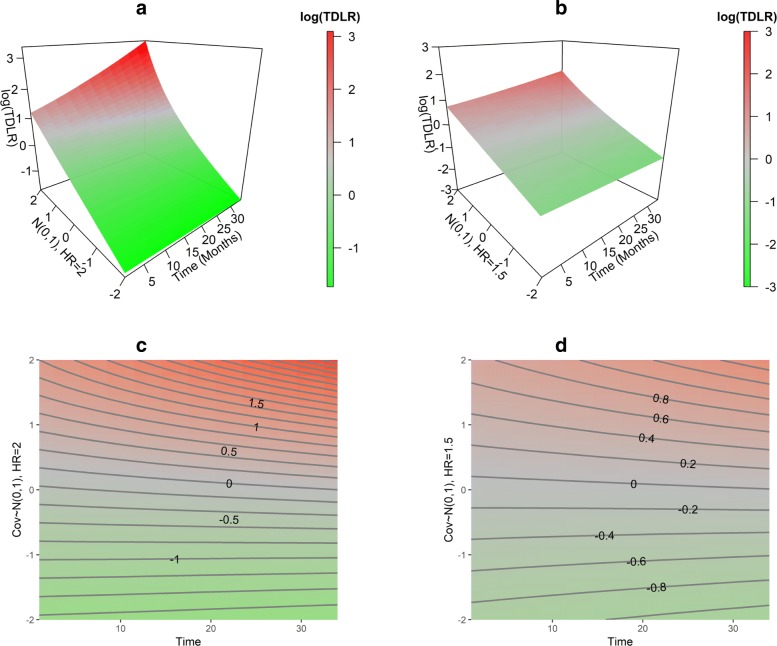
Simulated log TD-LR surfaces (1**a**, 1**b**) and contour plots (1**c**, 1**d**). Simulated log TD-LR surfaces or contours for covariates with HRs of 2 (1**a** and 1**c**) and 1.5 (1**b** and 1**d**)

Findings for simulations using three different sample sizes are very similar. The results for n=300 are presented in Fig. 1. Log-transformed *TD*−*LR*(*x,t*) value of 0 (gray regions indicated on the color scale) correspond to raw *TD*−*LR*(*x,t*) values of 1, indicating that at the given time point *t*, the marker value *x* provides no update to the KM-based estimate of the odds of event at or before *t*. A flat surface at 0 thus represents a marker that is uninformative: incorporating marker values into event risk estimates yields no update relative to previous KM-based estimates that do not incorporate marker information.

Positive (red colors) and negative (green colors) log*TD*−*LR*(*x,t*) respectively correspond to upward and downward adjustments of the KM-based estimate of odds of event at or before *t* due to the marker value *x*. A sloped surface with color changes (e.g., Fig. 1a, b) indicates that incorporating marker values does update KM-based estimates of event odds, and thus the marker is informative. Additionally, the larger HR yields a more steeply sloped surface with more curvature than does the smaller HR.

More specifically, examining cross-sections of the TD-LR surface at points on the *t*- and *x*-axes can further characterize the marker’s update to the odds of the event. At a fixed *t*, a positive slope for increasing *x* with color changes from cool (green) to warm (red) indicates that relative to the KM estimate of odds, individuals with lower marker values have lower odds of event at or before *t* and individuals with higher marker values have higher odds of event at or before *t*. A TD-LR surface with drastic changes in its color suggests a more informative marker.

When a marker value *x* is fixed, a positive slope (represented with a change from cooler to warmer colors) for increasing *t* indicates that the update from the marker value *x* is larger for later events, i.e., the likelihood of experiencing the event for individuals with this marker value increases with time. Similarly, a negative slope for increasing *t* at a fixed *x* indicates that the likelihood of event for individuals with this marker value decreases with time as seen for extreme marker values in Fig. 1a such that for (extreme) large marker values (e.g., *x*≈2), the surface bends upward for increasing *t* while for (extreme) small marker values (e.g. *x*≈−2), the surface bends downward. Markers exhibiting these patterns in log TD-LR values will yield surfaces that torque at later time points *t*. Alternatively, one can also present TD-LR as a contour plot to potentially to simplify observing subsections of the three-dimensional surface. See Fig. 1c and d.

To explore how much TD-LR estimates relied on the proportional hazard (PH) assumption. We have conducted additional simulation studies to examine the impacts of sample size, censoring percentage and PH assumption on the performance of TD-LR estimates in terms of bias and mean squared error (MSE) where the true TD-LR is computed using the theoretical values (for the marginal odds of *S*(*t*)) and numeric integration (for the conditional odds of *S*(*t*∣*x*)). The results of those simulation studies are summarized in Table 1. Briefly, under the PH situation, TD-LR estimates perform well and with a larger sample size, smaller censoring proportions in general are associated with smaller bias and MSE but the performance of TD-LR estimates at later time point (E.g., *t*=8) and/or at larger covariate values (e.g., *x*=1) are associated with much greater bias and MSE. This is expected because there are significantly fewer data points available either because many already had the event or have been censored for estimation at those (*t,x*) combinations even more so when the censoring percentage is high. This phenomenon is more exacerbated in the case when PH assumption is violated as we have expected. Furthermore, as shown by the histograms in “Appendix” section, simulations with sample sizes increasing up to 1000 suggested the asymptotic distribution of TD-LR is approximately normal at varying marker values and time points.

**Table 1:** Performance of TD-LR under the proportional and non-proportional hazards situations with various sample sizes, censoring percentage, time points, and marker values

			Proportional Hazard (PH)					
			*x*=-1		*x*=0		*x*=1	
N	Censoring		2 yr	8 yr	2 yr	8 yr	2 yr	8 yr
100	10-15%	Bias	-0.0034	-0.0218	-0.0118	-0.1755	-0.0410	-6.747
		MSE	0.0053	0.0060	0.0103	1.757	1.031	5.6×10^7^
	60-80%	Bias	-0.0061	-0.0074	-0.0085	-0.0098	-0.0434	-0.0444
		MSE	0.0164	0.0141	0.0132	0.0102	0.0448	0.1669
500	10-15%	Bias	-0.0023	-0.0035	-0.0029	-0.0226	-0.0126	2.729
		MSE	0.0010	0.0010	0.0016	0.1587	0.1275	3634
	60-80%	Bias	0.0066	0.005	0.0028	0.0010	-0.0084	-0.0177
		MSE	0.0033	0.0028	0.0026	0.0019	0.0082	0.0315
1000	10-15%	Bias	0.0013	-0.0021	-0.0006	-0.0248	-0.0169	-2.286
		MSE	0.0005	0.0005	0.0009	0.0760	0.0654	1042
	60-80%	Bias	0.0030	0.0030	0.0013	0.0013	-0.0071	-0.0116
		MSE	0.0015	0.0013	0.0012	0.0009	0.0041	0.0156
			Non-Proportional Hazard (PH), *γ*=0.2					
			*x*=-1		*x*=0		*x*=1	
N	Censoring		2 yr	8 yr	2 yr	8 yr	2 yr	8 yr
100	10-15%	Bias	0.0257	0.1984	-0.0749	-5.774	-2.542	−2.01×10^13^
		MSE	0.0036	0.0430	0.0198	33.84	11.16	4.02×10^26^
	60-80%	Bias	0.0324	0.0949	-0.0020	0.1766	-27.64	−2.95×10^64^
		MSE	0.0018	0.0103	0.0103	0.0509	766.8	8.72×10^128^
500	10-15%	Bias	0.0275	0.2080	-0.0669	-5.744	-2.87	−2.01×10^13^
		MSE	0.0013	0.0439	0.0068	33.06	8.664	4.02×10^26^
	60-80%	Bias	0.0324	0.0984	0.0046	0.2033	-27.98	−2.95×10^64^
		MSE	0.0012	0.0099	0.0021	0.045	783.2	8.72×10^128^
1000	10-15%	Bias	0.0263	0.2082	-0.069	-5.742	-2.89	−2.01×10^13^
		MSE	0.0010	0.0437	0.0060	33.01	8.597	4.02×10^26^
	60-80%	Bias	0.0328	0.0997	0.0076	0.2128	-27.99	−2.95×10^64^
		MSE	0.0012	0.0101	0.0014	0.0474	783.8	8.72×10^128^

^*^ *γ* as the coefficient for the interaction between time and marker value *x*. The summary was based on 500 simulation replicates

### SWOG 8710 Example

The data from SWOG 8710 comprise 231 individuals, of whom 172 died while under study. A secondary analysis of the SWOG 8710 data was interested in the neutrophil-lymphocyte ratio (NLR) as a prognostic marker for overall survival (OS), as the NLR is particularly easy to measure and had been found to be an independent prognostic factor in some studies [23–25].

Previous work had focused on treating the continuous NLR as a binary variable dichotomized as being above or below a certain cutoff, above which individuals were more likely to experience events and below which they were less likely to experience events. We sought to adhere to recommendations in analysis guidelines such as REMARK which cautions against the common clinical research practice of dichotomizing continuous markers due to the potential for bias and loss of information [26, 27]. Thus, we analyze NLR as a continuous marker in the current analysis.

**Fig. 2:**
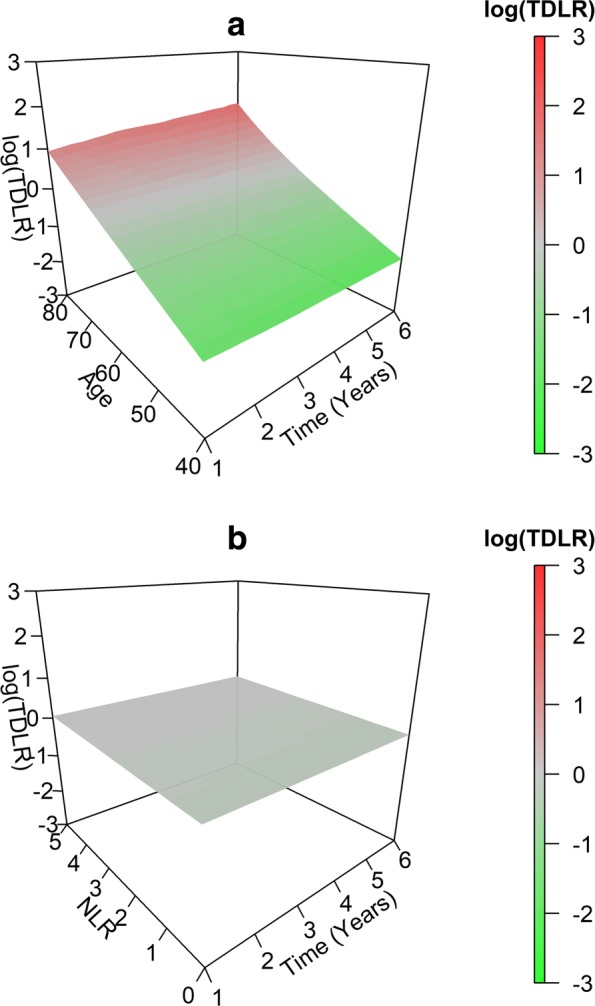
SWOG data TD-LR surfaces. Real data log TD-LR surfaces for age (2**a**) and NLR (2**b**)

**Fig. 3:**
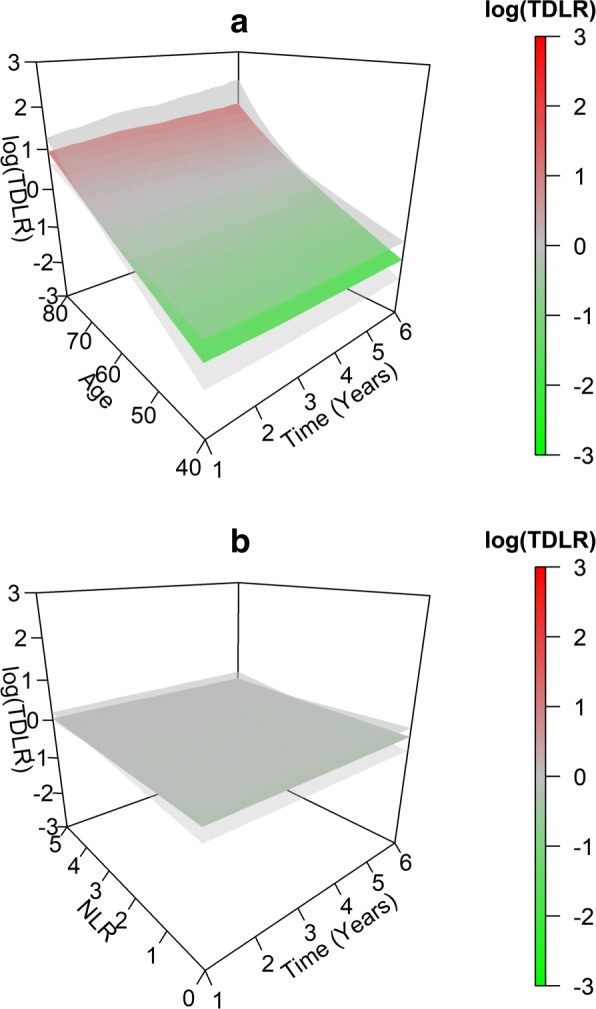
SWOG data TD-LR surfaces with bootstrap CIs. Real data log TD-LR surfaces for age (3**a**) and NLR (3**b**) with overlaid 95% bootstrap CIs

We illustrate the TD-LR surface estimation and visualization techniques using data from the SWOG 8710 clinical trial. Figure 2a and b present the estimated TD-LR surfaces for NLR (mean (SD) 3.3 (2.02), range 0.8–15.4) and age at randomization (mean (SD) 62.6 (9.04), range 36.9–84.1). We select age in this analysis because age was previously found to be an independent prognostic factor for overall survival in this data [2, 3]. In addition, neither NLR nor age (nor any other covariates considered in a subsequent analysis) were found to violate the proportional hazards assumption. Ojerholm et al. [28] Fig. 3a and b present the same surfaces with overlaid 95% bootstrap confidence intervals constructed from 1000 bootstrap resamplings.

The TD-LR surface for age is shown to have a moderate positive slope for both increasing marker value *x* and increasing *t*. After incorporating age, younger individuals (aged 40–50) have lower odds of death at or before *t* across all *t* relative to “baseline” or prevalence-based odds as captured by KM-based estimates. For 1<*t*<6 years, log TD-LR estimates for these younger individuals range between -1.5 and -0.9, which amount to multiplicative updates to KM-based odds of death at or before *t* of approximately 0.22 to 0.41. Similarly, TD-LR values for older individuals (aged 70–80) in the same time range vary between 0.3 and 1.5, amounting to multiplicative updates to KM-based odds of 1.4 to 4.5. For later *t* and older age, these multiplicative updates increase.

In contrast, the surface for NLR is close to zero across all marker values and time points (log TD-LR ranging from approximately -0.14 to 0.07), indicating the NLR provided little or no update to KM estimates of odds of death at or before *t* at any *t* in the range examined. The NLR marker does not appear to provide any informative update to existing estimates of odds of death.

To determine how much better age is than NLR at updating the KM-based odds estimates or to make direct comparisons at certain marker values, we implement the scale-invariant TD-LR, as the scales of age and NLR differ dramatically. The following analysis used landmark times *s*=0,1,2,5, and 10, and scale-invariant TD-LR estimates are calculated for *t*=*s*+2. We selected these time points because they are standardly used for bladder cancer research given its known natural history, e.g., 1 year captures very aggressive tumor related and treatment related mortality, 2 year captures about half tumor related mortality, 5 year captures almost all, 10 year captures non-tumor related mortality.

**Fig. 4:**
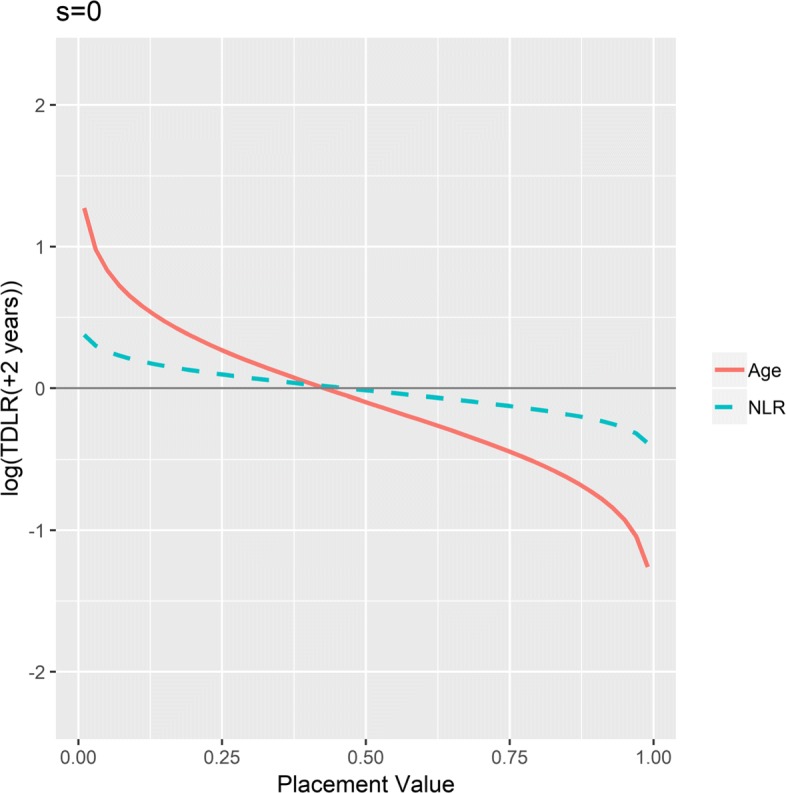
Scale-invariant TD-LR for age and NLR at *s*=0,*t*=2. Scale-invariant log TD-LR surface cross sections for age and NLR at landmark time 0 and time equal to +2 years

Figure 4 presents overlaid cross-sections of scale-invariant TD-LR surfaces for age and NLR at the first landmark time *s*=0 and *t*=2. The curvature of the cross-section for age suggests that for all marker values (on the placement value scale, or, equivalently, percentile scale), age provides larger (upward or downward) adjustment to odds of death at or before two years after the landmark time than do NLR values of the same percentiles. Thus, age is a more informative marker than NLR. Therefore, under the first landmark time *s*=0, based on all patients at risk for the following two years, age at its 75th percentile for example is associated with a scale-invariant TD-LR of exp(0.269)=1.309 compared to exp(0.098)=1.103 for NLR at its 75th percentile.

**Fig. 5:**
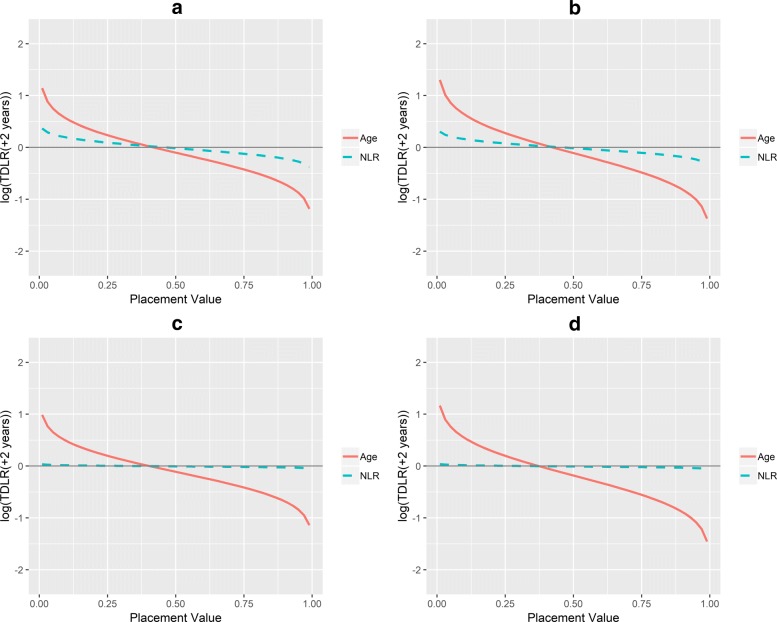
Scale-invariant TD-LR for age and NLR at various landmarks. Scale-invariant log TD-LR surface cross sections for age and NLR at landmark times 1,2,5,10 and time equal to +2 years

Figure 5 presents similar cross sections for the other landmark times (*s*=1,2,5,10). Similar to the observations at *s*=0, for small placement values, corresponding to high percentiles of age and NLR at the landmark times, the scale-invariant log(TD-LR) for age at 2 years after the landmark time is more positive than it is for NLR. For large placement values, corresponding to low percentiles of age and NLR at the landmark times *s*, the scale-invariant log(TD-LR) at 2 years after the landmark time for age is more negative than it is for NLR. Thus, age provides larger updates to KM-based odds of death than does NLR. Indeed, for later landmarks (Figure 5c and d), NLR appears to provide no update at all. NLR may not be a valuable marker for determining risk of death at these landmark times. We can also examine different time points after selected landmark times (e.g., 3 or 5 years after) to obtain a fuller understanding of trends in the scale-invariant TD-LR.

**Fig. 6:**
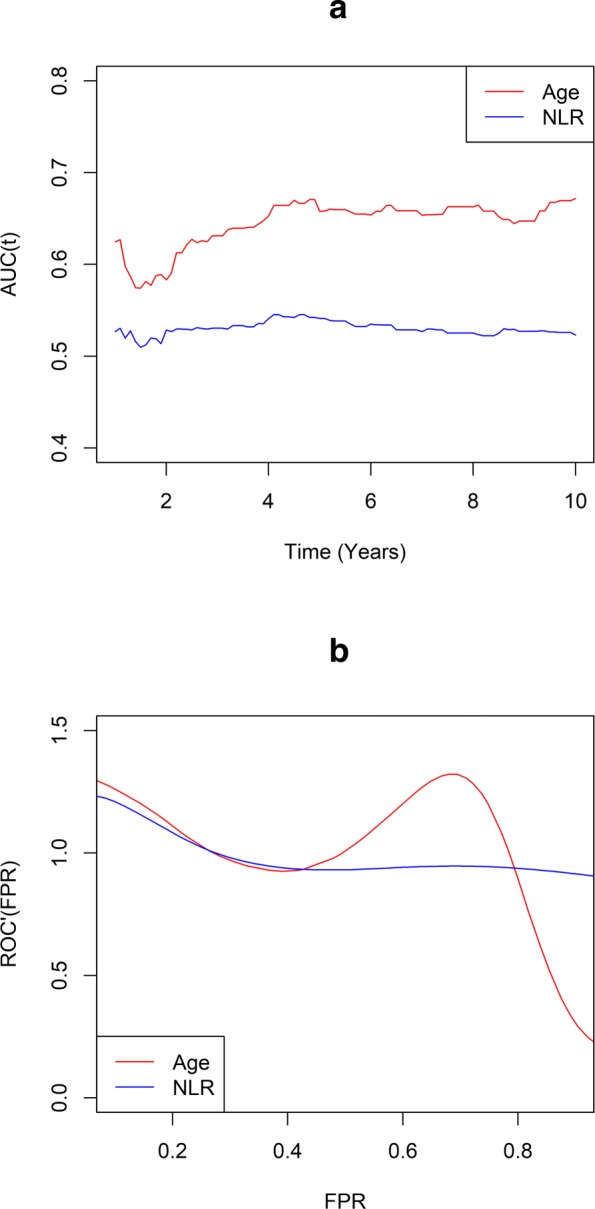
The relationships between TD-ROC and TD-LR. Estimates of TD-AUC(*t*) for age and NLR over time (6a) and derivatives of TD-ROC versus false positive rate

To illustrate the relationships between TD-ROC and TD-LR, we computed the area under the TD-ROC using the R package *survivalROC* for SWOG 8710 data. As shown in Fig. 6b, area under the TD-ROC for age is consistently higher than NLR for all *t*. We then plotted in Fig. 6b the smoothed curve for the derivative of TD-ROC at *t*=2 showing the value of *ROC*^′^(*t*=2) for age is greater than that of NLR for small placement values (FPR), and lower for large placement values. Thus based on our discussion on page 6, such pattern also implied that AUC(*t*=2) for age is greater than AUC(*t*=2) for NLR.

## Discussion and Conclusion

This work extends existing LR measures to understand the predictive ability of continuous markers from binary event data to survival data. The proposed time-dependent LR is estimated by a function of Cox PH and KM survival estimates. We chose these estimation methods for their ease of handling censoring, implementation, and desirable properties with respect to consistency. Additional work would be to more fully characterize the asymptotic distribution of $\widehat {TD- LR(x,t)}$.

The use of Cox PH and KM survival estimates means that the updates quantified by *TD*−*LR*(*x,t*) here are with respect to a prevalence-based estimate of odds of event at or before *t*. However, our estimation approach could easily be modified to define the “baseline” estimate of odds of event at or before *t* using survival probability estimates from a Cox PH model that incorporates existing or known prognostic factors. The TD-LR in this extended case would represent the update to the existing estimate of odds of event at or before *t* obtained from incorporating a new marker *X* in addition to the other factors. To present the TD-LR as a three dimensional surface, one would first need to fix every predictor other than the marker at specific values, and the TD-LR surface would thus be estimated for a specific covariate profile. Potentially, the proposed approach can be extended to consider more than one new markers at the same time, although the TD-LR surface will be difficult to interpret.

Comparison of markers with respect to the estimated TD-LR values is accomplished by standardizing the markers through the placement value. Survival probability estimates needed for the TD-LR estimates for placement values cannot be calculated directly because placement value is considered as an internal time-dependent covariate. Our current solution is to apply landmark analysis methods. The landmark analysis method, though simple, does not provide a complete picture of the relationship between placement value and survival probabilities. Potential solutions include redefining the reference population used to calculate placement value, such that its calculation does not depend on the survival behavior of the individuals under study.

The candidate marker itself may be time-dependent. For internal time-dependent markers, landmark analysis, or other techniques that allow survival probability estimation conditional on values of internal time-dependent covariates, will be required. For external time-dependent markers, regular time-dependent covariate estimation techniques can be used. Similarly, the effect of the candidate marker on hazard of event obtained in the Cox PH estimation may be time-dependent. In this case, techniques for estimating time-dependent coefficients in Cox PH regression could be employed to estimate the TD-LR.

We note the relationship between the TD-LR and the derivative of the TD-ROC curve to illustrate comparison of time-dependent AUC can also be achieved by using the TD-LR. While many guidelines such as REMARK caution against the dichotomization of continuous markers, if the determination of an “optimal cutoff” for clinical use is desired, TD-LR can also be used for this purpose. For example, one commonly used approach to identify an optimal cut-off is to find the marker value that gives the shortest distance from a ROC or TD-ROC curve to the top left corner of (0,1) (i.e., 0% false positive rate and 100% true positive rate). The concave nature of ROC or TD-ROC curves suggests that the point on the curve that minimizes this distance should occur where the TD-ROC’ is approximately equal to 1. Therefore, a cutoff point for a given TD-ROC curve could also be determined through TD-LR alone. The optimal cutoff value (or ranges of possible cut-off values) across different time points could be determined by finding the marker value that yields a TD-LR of approximately 1 across the most time points that relevant to specific clinical applications.

Current challenges for TD-LR estimation center around incorporating more complex time dependence to both markers and other predictors, as well as adapting the estimation methods to the case of competing events. Future work might readily address the latter issue by using cause-specific Cox models or Fine-Gray models, though extending these methods to the TD-LR will require care to ensure correct consideration of individuals who do not experience the event of interest or who have a competing event.

The LR is an extant method for measuring updates to risk prediction due to the knowledge of a (binary or continuous) marker value, but its use has not been explored in censored survival data. The TD-LR described in this work can provide a richer understanding of how continuous markers relate to the likelihood of events in censored survival data contexts through the use of an easily interpretable three-dimensional surface. This graphical display may aid in communication with clinical audiences that, despite being accustomed to using measures like the HR, may appreciate the simplicity of the TD-LR. Comparison of predictive ability across continuous markers, even when marker scales differ greatly, is also enabled by the proposed techniques. One point to keep in mind is that the calculation of TD-LR is relied on proportional hazards assumption to be valid in order to use Cox model to estimate the conditional odds at a time point and a marker value, and have enough observations available for the time points and marker values that one wish to explore. Thus we recommend to check the proportional hazard (PH) assumption first and the ranges of the data values before proceeding with the estimation. If PH assumption is not appropriate, alternative models such as parametric regression can be used instead of a Cox model.

## Appendix

**Fig. 7:**
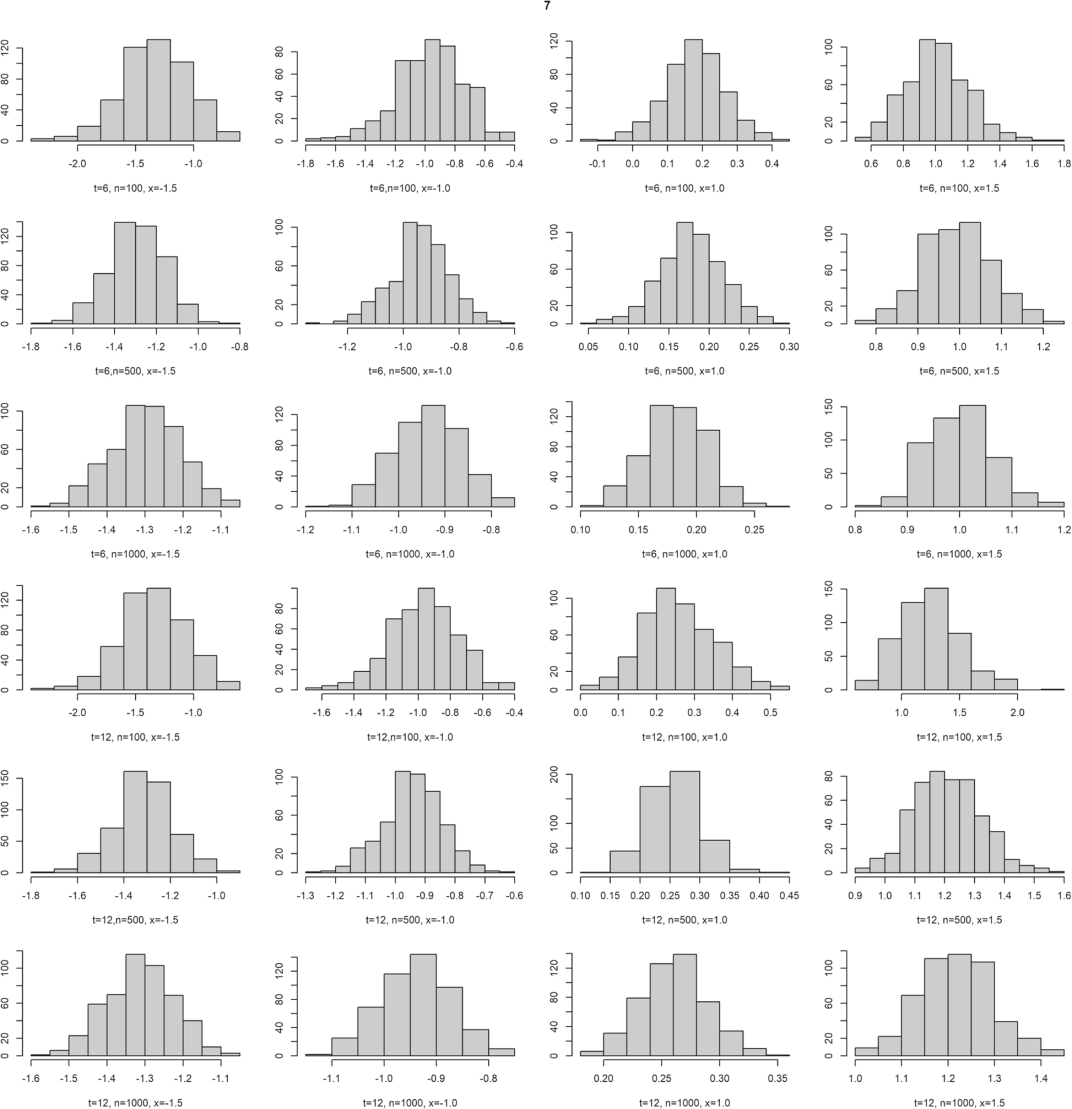
Histograms of estimated TD-LR. Sample sizes (100, 500, 1000), time points (*t*=6,12), and marker values (*x*=−1.5,−1,1,1.5). The hazard ratio for the marker value is assumed to be 2

## Abbreviations

AUC: Area under the curve
HR: Hazard ratio
KM: Kaplan-Meier
LR: Likelihood ratio
NLR: Neutrophil-to-lymphocyte ratio
PH: Proportional hazards
ROC: Receiver-operator characteristic
SWOG: Southwest Oncology Group
TD-LR: Time-dependent likelihood ratio
TD-ROC: Time dependent receiver-operator characteristic
